# Kinesin family member 2A acts as a potential prognostic marker and treatment target via interaction with PI3K/AKT and RhoA/ROCK pathways in acute myeloid leukemia

**DOI:** 10.3892/or.2022.8463

**Published:** 2022-12-13

**Authors:** Xinglin Liang, Ruixiang Xia

Oncol Rep 47: 18, 2022; DOI: 10.3892/or.2021.8229

Following the publication of the above article, the authors have realized that an error was made during the compilation of Fig. 9, as it appears on p. 10; essentially, the β-actin bands featured in Fig. 9A were inadvertently copied across to Fig. 9B.

The revised version of Fig. 9, now showing the correct β-actin bands for Fig. 9B, is shown on the next page. All the authors approve of the publication of this corrigendum, and the authors are grateful to the Editor of *Oncology Reports* for granting them the opportunity to publish this. The authors regret their oversight in allowing this error to be included in the published paper, and apologize to the readership for any inconvenience caused.

## Figures and Tables

**Figure 9.**
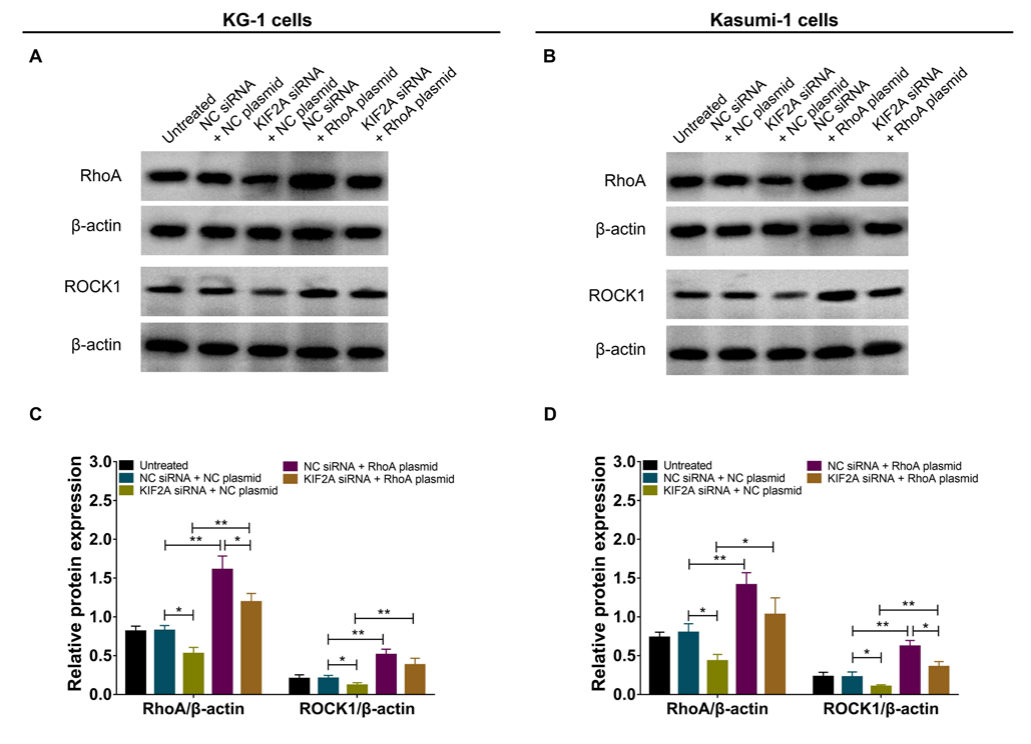
Successful transfection of RhoA overexpression plasmid in AML cells. Western blot analysis of (A) KG-1 and (B) Kasumi-1 cells following RhoA overexpression. RhoA and ROCK1 protein expression levels in (C) KG-1 and (D) Kasumi-1 cells following RhoA overexpression. *P<0.05, **P<0.01. RhoA, ras homolog family member A; AML, acute myeloid leukemia; ROCK1, Rho associated coiled-coil containing protein kinase 1; NC, negative control; KIF2A, kinesin family member 2A; si, small interfering.

